# Chapin A. Harris' Principles and Practice of Dentistry

**Published:** 1885-01

**Authors:** 


### BIBLIOGRAPHICAL.

HARRIS' PRINCIPLES AND PRACTICE OF DENTISTRY.—Including Anatomy, Physiology, Pathology, Therapeutics, Dental Surgery and Mechanism. Revised and edited by Ferdinand J. S. Gorgas, A. M., M. D., D. D. S., Author of "Dental Medicine," Editor of "Harris' Dictionary of Medical Terminology and Dental Surgery," Professor of Dental Science, Dental Surgery, etc., University of Maryland. Publishers: Blakiston, Son, & Co., Philadelphia, 1885, Eleventh Edition. Pages: 994. Illustrations: 740.

The first edition of Chapin A. Harris' "Principles and Practice of Dentistry" was published in 1841, and from that date it has been the principal text-book in all dental schools.

The last or tenth revision was issued under the careful supervision of the late Professor Philip H. Austen, M. D., D. D. S., assisted, in the parts relating to anatomy and physiology, by Dr. Thos. S. Latimer, and in parts relating to pathology and surgery, by the editor of the present edition. As the ten years prior to this revision had nearly revolutionized dental mechanism, Professor Austen found it necessary to almost re-write the portion of the work relating to "Mechanics," and its superior excellence was universally acknowledged.

Nearly fourteen years having elapsed since this was done, the rapid advances made during this period in Dental Histology, Pathology, Surgery, and also, to a considerable degree, in Mechanism, have necessitated another revision, and at the request of the author's family, and of the publishers, the editor has alone undertaken the task of revision, and the present edition is the result of more than a year's labor. This duty has been assumed with the hope that an experience of over a quarter of a century as a teacher in dental schools, and also as a dental practitioner, may have furnished the qualifications for such an undertaking.

The time which has elapsed since the first appearance of the tenth edition has necessitated a greater revision of this work than has been the case with any former edition, and the task of preparing an entirely new work would have been no greater.

Considerable changes have been made in the general arrangement of subjects ; a number of entirely new chapters have been added in the consideration of subjects not even alluded to in former editions ; additions have also been made to the text of nearly every chapter, some of the latter being far in excess of the original text.

The number of illustrations has been greatly increased, and the new matter now inserted has brought the work fully up to the time of its publication.

Obsolete theories and processes, together with unimportant details, have been omitted, and more useful matter substituted. The aim of the editor has been to meet the demands of the present advanced state of dental science.

The new matter added includes: The Development of the Bones of the Head and Face; Temporo-Maxillary Articulation: Description of Mucous Membrane; The Origin and Development of the Teeth; Analyses of Tooth Structures; Secondary Dentine; Dentition; Calcification and Decalcification of the Teeth; Alveolar Pyorrhœa; Apthous Stomatitis; Thrush; Sanguinary Calculus; Malformed Teeth; Effects of Syphilis upon the Dental Structures; Sensitive Dentine; Theories as to the Cause of Dental Caries; Treatment of Dental Caries; New Methods, Materials and Instruments Employed in Filling Teeth and other Operations; Electric Mouth Lamp; Electric Mallet; Dental Engines and Attachments; Rubber Dam Appliances; Treatment and Appliances for Correcting Irregularity of the Teeth; Contour Fillings; Replantation and Transplantation of Teeth; Different Methods of Inserting Artificial Crowns on Natural Roots; Bridge Work; General & Local Anæsthetic Agents; Improved Forceps; New Materials and Trays for Impressions; Articulators; Blowpipes; Furnaces; Celluloid; New Apparatus for Vulcanizing Rubber and Moulding Celluloid; Repairing Vulcanite; Duplicating Dentures; Theory of Vulcanizing; Regulators; Zylonite; Gold Alloy and other Cast Bases: Temperament in Relation to Natural and Artificial Teeth; Improvements in Porcelain Teeth; New Splints for Fracture of the Jaws; etc.; etc.; etc.

The Eleventh Edition of Harris' "Principles and Practice of Dentistry" is submitted to the profession, with a hope that it will be found a useful elementary treatise, a text-book for the student, and a reliable guide for the dental practitioner.

